# Letter to the Editor From de Zegher and Ibáñez: “Distinct Reproductive Phenotypes Segregate With Differences in Body Weight in Adolescent Polycystic Ovary Syndrome”

**DOI:** 10.1210/jendso/bvae074

**Published:** 2024-05-08

**Authors:** Francis de Zegher, Lourdes Ibáñez

**Affiliations:** Leuven Research & Development, University of Leuven, 3000 Leuven, Belgium; Endocrinology Department, Institut de Recerca Sant Joan de Déu, University of Barcelona, 08950 Esplugues, Barcelona, Spain; Centro de Investigación Biomédica en Red de Diabetes y Enfermedades Metabólicas Asociadas (CIBERDEM), Instituto de Salud Carlos III, 28029 Madrid, Spain

**Keywords:** PCOS, adolescence, androgen excess, oligo-anovulation, ectopic fat, oral contraceptives

Chen-Patterson et al (of the CALICO consortium) [1] are to be complimented for describing the contemporary phenotype of polycystic ovary syndrome (PCOS) in adolescent girls (age range, 14-18 years) across the United States, and for unravelling how PCOS features may differ between adolescents with a body mass index (BMI) below the 85th percentile (“lean”) vs those with a BMI above the 95th percentile (“with obesity”) [1]. The relevance of this paper can be raised by elaborating on its epidemiological, developmental, and therapeutic implications.

By definition, there are supposed to be 17-fold more adolescent girls with a BMI below the 85th percentile than with a BMI above the 95th percentile. In the CALICO database, however, the PCOS cases with obesity outnumbered the lean PCOS cases more than 4-fold, namely 4.5-fold (373 vs 82) before the exclusion of patients on medications, and 4.2-fold (286 vs 68) after their exclusion [1]. These unique numbers suggest that girls with obesity are approximately at a 75-fold higher risk of developing adolescent PCOS than lean girls. Such obesity-linked risk fits into the emerging concept that adolescent PCOS is essentially driven by ectopic fat and/or insulin resistance, both of which may ensue from a mismatch between the capacity and the demand to store lipids in subcutaneous adipose tissue [2, 3].

Under “data collection,” Chen-Patterson et al mention that “medical information included birthweight” but they appear to share no results on this variable [1]. Birthweight data from lean girls with adolescent PCOS would be welcome, given that their average weight at term birth may have been below the mean for girls in a contemporary US cohort [2, 3]. Birthweight data from adolescents with PCOS and obesity would also be welcome, given that these girls are at an 18-fold higher risk of developing type 2 diabetes [4], and that such development depends on interactions among genetic risk, prenatal weight gain, and postnatal BMI [5].

Chen-Patterson et al state that “combined oral contraceptive pills remain the first-line pharmacological treatment for PCOS based on current guidelines,” and that their identification of distinct subgroups within adolescent PCOS provides a rationale “for individualized treatment approaches” [1]. Distinct approaches are still under investigation (reviewed in [3]) but pilot evidence suggests that medications targeting a loss of ectopic fat and/or a gain of insulin sensitivity are capable of reversing the adolescent PCOS phenotype [6, 7]. The first results are so promising that they may even suffice to concur that regulatory agencies should refrain from approving combined oral contraceptive pills as the first-line pharmacological treatment for adolescent PCOS, certainly in lean girls [3, 6].

## Disclosures

The authors have nothing to disclose.

## Abbreviations

BMI: body mass index
PCOS: polycystic ovary syndrome
